# A Sensitive Carbon Dioxide Sensor Based on Photoacoustic Spectroscopy with a Fixed Wavelength Quantum Cascade Laser

**DOI:** 10.3390/s19194187

**Published:** 2019-09-26

**Authors:** Shunda Qiao, Yanchen Qu, Yufei Ma, Ying He, Yao Wang, Yinqiu Hu, Xin Yu, Zhonghua Zhang, Frank K. Tittel

**Affiliations:** 1 National Key Laboratory of Science and Technology on Tunable Laser, Harbin Institute of Technology, Harbin 150001, China; 18S021047@stu.hit.edu.cn (S.Q.); quyanchen@hit.edu.cn (Y.Q.); hearkenyi@hit.edu.cn (Y.H.); 1162100203@hit.edu.cn (Y.W.); 1162100119@stu.hit.edu.cn (Y.H.); yuxin0306@hit.edu.cn (X.Y.); zhzhang@hit.edu.cn (Z.Z.); 2 Department of Electrical and Computer Engineering, Rice University, 6100 Main Street, Houston, TX 77005, USA; fkt@rice.edu

**Keywords:** photoacoustic spectroscopy, carbon dioxide detection, quantum cascade laser

## Abstract

A photoacoustic spectroscopy (PAS) based carbon dioxide (CO_2_) sensor with a fixed wavelength quantum cascade laser (FW-QCL) was demonstrated. The emission wavelength of the FW-QCL at 4.42 μm in the mid-infrared spectral region matched a fundamental CO_2_ absorption line. Amplitude modulation of the laser intensity was used to match the resonant photoacoustic (PA) cell. The noise from the background was reduced with the correlation demodulation technique. The experimental results showed that the sensor had excellent signal stability and a concentration linear response. When the integration time was 1 s, a 1σ minimum detection limit (MDL) of 2.84 parts per million (ppm) for CO_2_ detection was achieved. The long-term stability of the sensor was evaluated by means of an Allan deviation analysis. With an integration time of ~100 s, the MDL was improved to 1 ppm. This sensor was also used to measure the CO_2_ concentration from some common emission sources, such as cigarette smoking, automobile exhaust, and the combustion of some carbon-containing materials, which confirmed the stability and robustness of the reported FW-QCL based CO_2_-PAS sensor system.

## 1. Introduction

Carbon dioxide (CO_2_) is a colorless and odorless gas and is one of the main greenhouse gases. With the increasing use of fossil fuel combustion and emissions from automobile exhaust, the CO_2_ concentration in the atmosphere has increased from 280 parts per million by volume (ppmv) to ~400 ppmv since the industrial revolution [1,2]. The greenhouse effect has led to global warming, glacier melting, and a rise in sea levels [3,4]. If the CO_2_ concentration increases even further, these phenomena will become worse and affect people’s life severely in the future. In addition, the high concentration of CO_2_ also reduces the pondus hydrogenii (PH) of oceans and soil which impacts marine organisms and plants [5,6]. Therefore, there is a need to develop a sensitive CO_2_ gas sensor to monitor CO_2_ concentrations.

In 1880, Bell reported the photoacoustic effect, when he discovered that when thin discs were irradiated with modulated light, an acoustic signal is produced [7]. Subsequently, many applications based on the photoacoustic effect were developed and photoacoustic spectroscopy (PAS) has become one of the most important examples [8,9,10]. PAS is widely used to detect trace gases because of its advantages, such as high detection sensitivity, fast response time, and a wide dynamic range [11,12,13,14,15]. The main principle of the PAS technique is to use a modulated laser to stimulate the target gas molecules. The gas molecules absorb the optical energy from the photons and are excited. Subsequently these molecules relax to the ground state by a non-radiative process which produces localized heat. This heat results in gas expansion, which increases the local pressure. With a modulated laser, the local temperature will change periodically and a pressure wave, which is the acoustic signal, is produced. This acoustic signal can be detected with a sensitive microphone detector and used to determine the gas concentration [16,17,18]. Many trace gases detection methods based on PAS have already been demonstrated [19,20,21,22,23,24,25,26,27].

The mid-infrared spectral region is the fundamental absorption spectral band for many molecules. This means that the absorption intensity in the mid-infrared region is the strongest in the entire absorption spectrum [28]. Therefore, a laser source with an emission wavelength located in the mid-infrared region improves the sensor’s detection sensitivity. A quantum cascade laser (QCL), which was invented in 1994 [29], is an attractive laser source for a trace gas sensing system based on PAS. The spectral range of a QCL can cover the entire mid-infrared region. Based on the luminescence mechanism of the QCL one electron can emit many photons, which is advantageous for producing a high optical power [30]. The commercial sensors based on QCL have the advantages of high selectivity compared with the ones based on thermal micro electro mechanical system (MEMS) emitters and non-resonant PA cell with MEMS microphones [31,32,33,34,35]. Therefore, the QCL based PAS sensors have excellent performance and application prospects [36]. 

In this paper, a sensitive PAS-based CO_2_ trace gas sensor was demonstrated. The excitation source was a fixed wavelength QCL (FW-QCL) emitting at 4.42 μm. A resonant photoacoustic (PA) cell together with a central cylinder tube was used as an acoustic resonator. Two buffers were placed at both sides of the cylinder to reduce the noise from the gas flow. The PAS signal was detected with a condenser microphone. In order to modulate the laser optical intensity a chopper was applied. A correlation demodulation technique was used in order to reduce the noise from the background. The long-term stability of the sensor system was evaluated with an Allan deviation analysis. Reliable and robust operation of the PAS sensor was demonstrated by continuous monitoring of atmospheric CO_2_ concentration levels. The sensor was also used to detect the CO_2_ concentration levels from cigarette smoking; automobile exhaust; and the combustion of paper, cable sheaths, as well as timber demonstrating that the reported mid-infrared sensor had an excellent operating performance with a potential in real world applications. 

## 2. Experimental Setup

### 2.1. CO_2_ Absorption Line Selection

**Figure 1:**
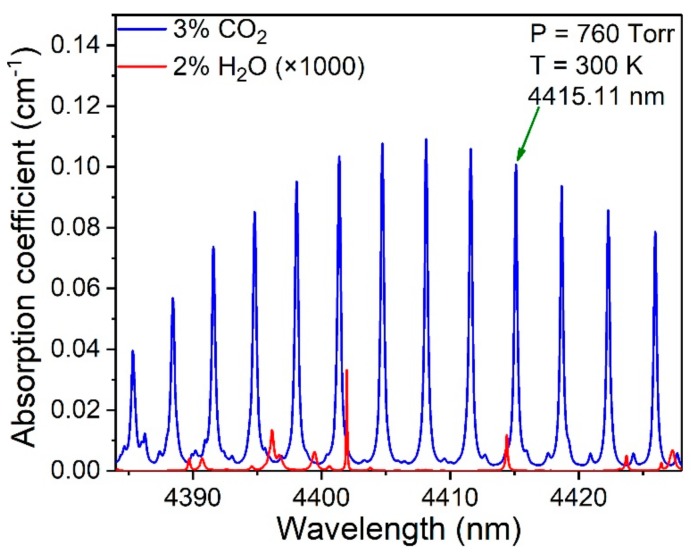
Absorption lines for 3% CO_2_ and 2% H_2_O in the ~4.4 μm spectral region based on the HITRAN 2016 database.

With the excellent tunability of the QCL in the mid-infrared region, it is possible to select a fundamental CO_2_ molecular absorption line. According to the HITRAN 2016 database [37], at a temperature of 300 K, the CO_2_ absorption lines located at ~4.4 μm at standard atmospheric pressure are shown in Figure 1. It can be seen that an absorption line located at a wavelength of 4415.11 nm (2264.95 cm^−1^) is one of the strong absorption lines. Furthermore, this line avoids H_2_O interference at ~4.4 μm.

### 2.2. QCL Performance Characteristics

**Figure 2:**
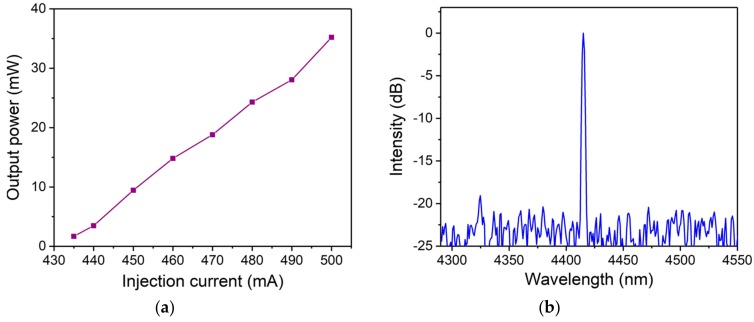
(**a**) The fixed wavelength quantum cascade laser (FW-QCL) output power as a function of at injection current. (**b**) The emission spectrum of the FW-QCL with a 35.2 mW optical power.

The excitation source of the CO_2_-PAS sensor system was a fixed wavelength QCL (FW-QCL, Model No. 41045-UF, Daylight Solutions), in which the laser was tuned to the desired wavelength by means of a diffraction grating. The output power of the FW-QCL can be increased by varying the injection current. The laser power as a function of injection current is shown in the Figure 2a. From this figure it can be seen that when the injection current was 500 mA, the output power was 35.2 mW. The wavelength of the FW-QCL was determined with a wavelength meter which has a resolution of 0.2 pm (Model No. 721A, Bristol). The results of these measurement are shown in Figure 2b. As shown in Figure 2b, the laser central wavelength was 4415.11 nm when the output power was 35.2 mW, which was a good match for the selected CO_2_ absorption line. The signal-to-noise ratio (SNR) of the laser emission spectrum was ~20 dB.

### 2.3. Sensor System Configuration

**Figure 3:**
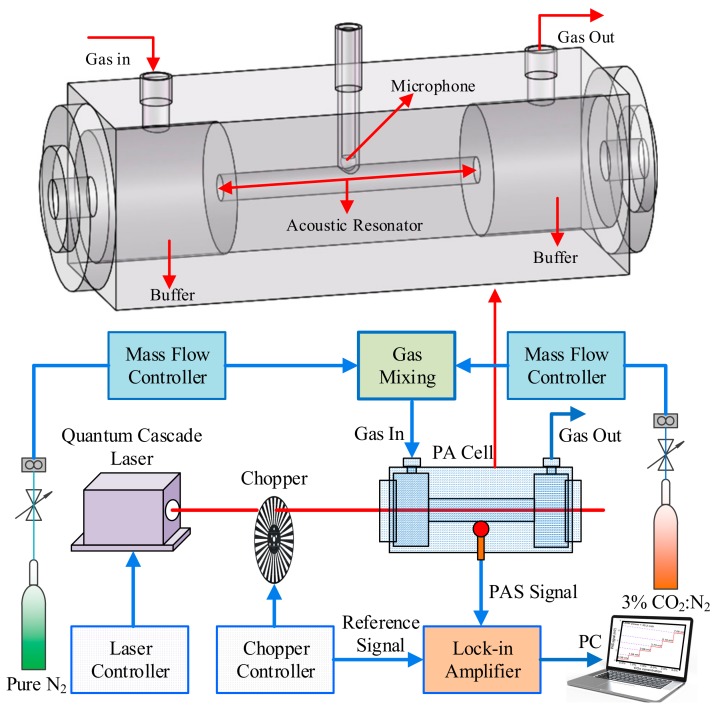
The Schematic of the photoacoustic spectroscopy (PAS) based CO_2_ sensor system with a FW-QCL.

The schematic of the PAS based CO_2_ sensor system is shown in Figure 3. A resonant PA cell was used in this system which had two calcium fluoride (CaF_2_) window mirrors and a central cylinder tube made of aluminum. This tube was used as the acoustic resonator and operated in a one-dimensional resonant mode. The resonant frequency was 1580 Hz. The radius and length of the acoustic resonator were 5 mm and 100 mm, respectively. To reduce the noise from the gas flow, two buffers were placed at both ends of the cylinder tube. The radius and length of the buffer were 25 mm and 50 mm, respectively. Therefore, the total length of the PA cell was 200 mm. A condenser microphone with detection sensitivity of 50 mV/Pa was placed inside the PA cell to detect the acoustic signal generated from the target gas. The FW-QCL operated in a continuous wave (CW) mode. A mechanical chopper was placed between the laser and PA cell to modulate the optical intensity. The modulation frequency of the chopper was set equal to the resonant frequency of the resonator to obtain the strongest PAS signal. In order to vary the target gas concentrations, two mass flow controllers with a mass flow uncertainty of 3% were used to dilute a 30,000 ppm CO_2_–N_2_ gas mixture with pure N_2_. The flow rate was controlled at 120 ml/min. The signal from the microphone was then sent to a lock-in amplifier. The lock-in amplifier employed a correlation demodulation technique to reduce the noise from the background. The constant time of the lock-in amplifier was 1 s. The final detected signal was displayed on a laptop computer using the lock-in amplifier software interface.

## 3. Results and Discussions

**Figure 4:**
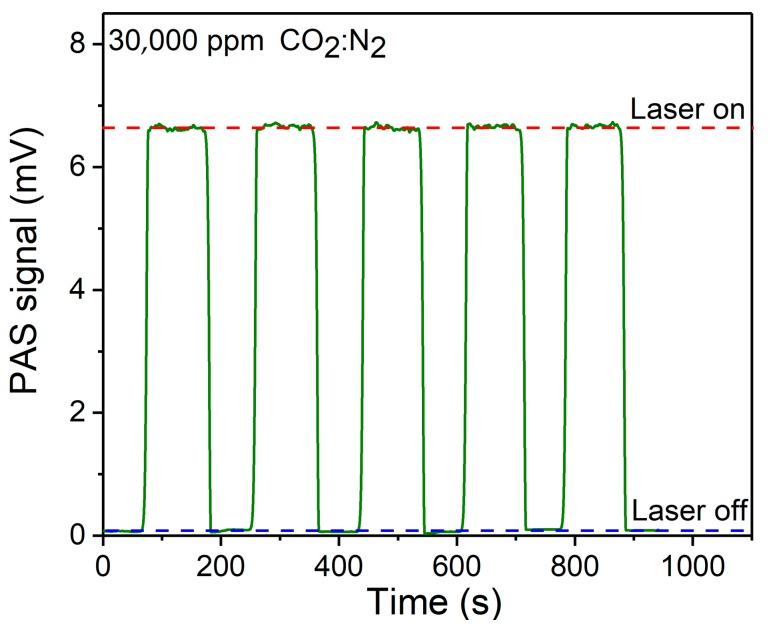
Signal stability of the reported FW-QCL based CO_2_ sensor system.

The signal stability of the CO_2_-PAS sensor system was tested. The modulation frequency of the chopper was set at 1580 Hz to match the resonant frequency of the PA cell. The optical power of the FW-QCL was 35.2 mW with an injection current of 500 mA. A 30,000 ppm gas mixture of CO_2_ and N_2_ was used. In order to test the stability of the sensor system, the PAS signal was detected with and without FW-QCL many times. The results of the tests are shown in Figure 4. It can be seen that with the same CO_2_ gas concentration, the signal amplitude of the CO_2_-PAS sensor could be maintained at the same level, which indicated an excellent sensor system stability.

**Figure 5:**
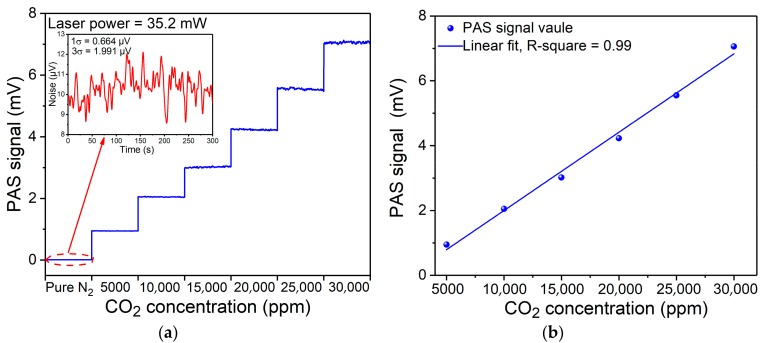
(**a**) System noise and PAS signal for different CO_2_ concentration levels. (**b**) PAS signal as a function of CO_2_ concentration.

In order to verify the concentration response of the PAS based sensor system with a FW-QCL, the PAS signal was detected with different CO_2_ concentrations. At a standard atmospheric pressure, a 30,000 ppm CO_2_–N_2_ mixture was diluted with pure N_2_ using two mass flow controllers. The CO_2_ detection results are depicted in Figure 5a. It can be seen that the CO_2_-PAS signal increased with an increase of the gas concentration. Furthermore, the noise of the sensor system was measured when the PA cell was filled with pure dry N_2_. From the inset of Figure 5a, it can be seen that with an integration time of 1 s, the 1σ background noise was 0.664 μV and the 3σ background noise was 1.991 μV. Therefore, the 1σ and 3σ minimum detection limit (MDL) of the PAS based sensor system with a fixed wavelength QCL for the detection of CO_2_ was 2.84 ppm and 8.46 ppm, respectively. The PAS signal as a function of CO_2_ concentration is shown in Figure 5b. After a linear fitting routine was used, the obtained R-square was ~0.99, which means that the sensor system has an excellent linear response to gas concentrations

**Figure 6:**
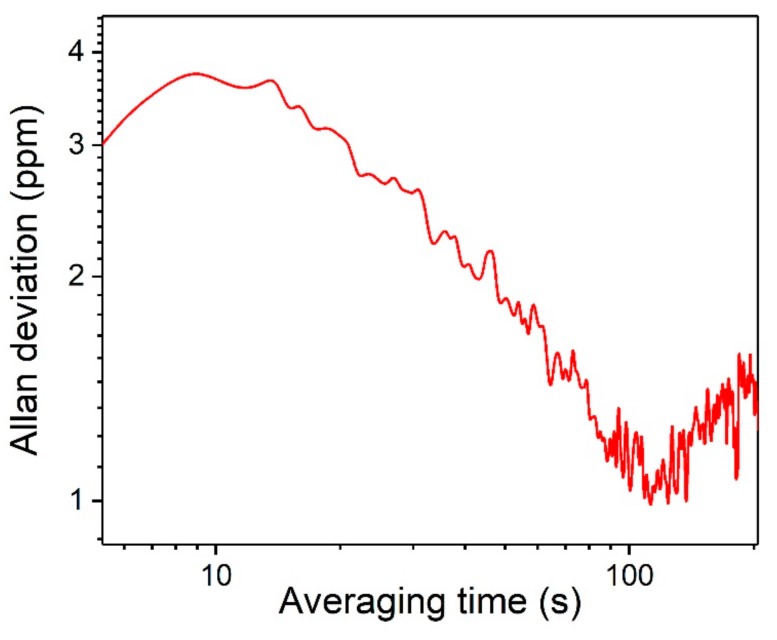
Allan deviation analysis for the FW-QCL based CO_2_ sensor system.

The long-term stability of the reported sensor system was evaluated by means of an Allan deviation analysis. The Allan deviation shows the relationship between the system stability and the averaging time. Pure dry N_2_ was flushed into the PA cell with a constant flow. The PAS signal was measured for a three hours period. The result of this measurement is shown in Figure 6, where it can be seen that when the integration time was ~100 s, a MDL of 1 ppm can be obtained. When the integration time was larger than 100 s system drifts start to dominate.

**Figure 7:**
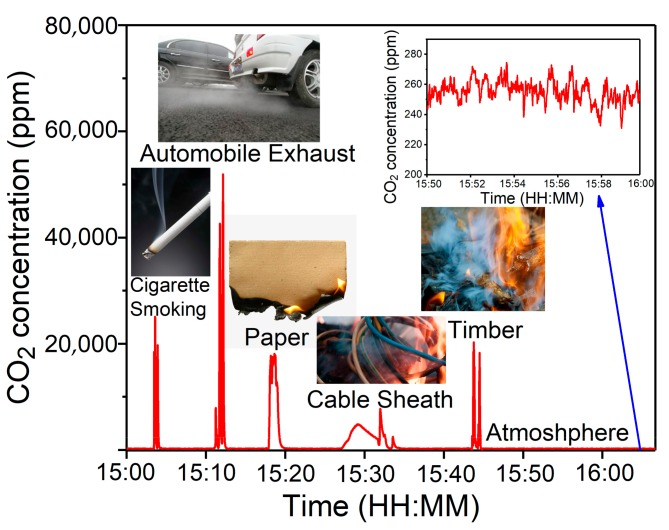
CO_2_ detection from several sources with the reported QCL based CO_2_ sensor system on the HIT campus, Harbin, China (Latitude and longitude are: 45°43′N/126°37′E).

There are many sources of CO_2_ emissions in daily life, such as cigarette smoking, the exhaust from automobiles and the combustion of carbon-containing materials. The CO_2_ concentrations from these sources were much higher than the MDL of the reported PAS based CO_2_ sensor system. Therefore, it is possible to detect the CO_2_ concentration from these sources with the reported sensor system. In the measurements, an inlet tube was placed outside the laboratory and the atmospheric air was pumped into the CO_2_-PAS sensor. The measured results are shown in Figure 7. It can be seen that the atmospheric CO_2_ concentration level was ~250 ppm and that the emission levels from automobile exhaust are the highest.

## 4. Conclusions

In conclusion, a PAS based CO_2_ sensor using a FW-QCL was demonstrated in this manuscript. The laser was tuned to the desired wavelength by using a diffraction grating. Such a fixed wavelength (FW) configuration has the merits of easy design and is advantageous for application of the reported sensor system. The emission wavelength of the FW-QCL was set at 4.42 μm in the mid-infrared region, which matched a fundamental CO_2_ absorption line and significantly improved the absorption intensity when compared to the overtone absorption band in the near-infrared region. A resonant PA cell with a resonant frequency of 1580 Hz was used to accumulate the acoustic energy. A mechanical chopper was employed to modulate the laser intensity. A correlation demodulation technique was used to reduce the noise from the background. When the integration time of the lock-in amplifier was set to 1 s, a 1σ MDL of 2.84 ppm for the detection of the CO_2_ was achieved. An Allan deviation analysis was applied to evaluate the long-term stability of the reported CO_2_ sensor. The results showed that when the integration time was ~100 s, a MDL of 1 ppm can be obtained. This sensor was also used to measure the CO_2_ concentration from several common emission sources, such as cigarette smoking, automobile exhaust and the combustion of some carbon-containing materials. These measurements indicated the stability and robustness of the reported FW-QCL based CO_2_-PAS sensor system. With a high detection sensitivity and stability, this FW-QCL based CO_2_-PAS sensor is suitable for applications in industrial processing, environmental monitoring, and fire detection.
